# Probabilistic functionalism as a limiting condition for robustness

**DOI:** 10.1038/s41598-025-32580-z

**Published:** 2025-12-22

**Authors:** Mattias Forsgren, Benjamin Mandl, Gustav Karreskog Rehbinder

**Affiliations:** 1 Department of Psychology, Uppsala University, P. O. Box 1225, 751 42 Uppsala, Sweden; 2 Department of Economics, Stockholm School of Economics, Stockholm, Sweden; 3 Verbund AG, Vienna, Austria; 4 Institute for Futures Studies, Stockholm, Sweden; 5 Department of Economics, Uppsala University, Uppsala, Sweden

**Keywords:** Probabilistic functionalism, Robustness, Attraction effect, Default nudge, Psychology, Human behaviour

## Abstract

When should we expect behavioural phenomena to be robust? We argue that many phenomena of interest to behavioural scientists, by their very nature, involve manipulations of stimulus characteristics. If there exist contingencies between those stimulus characteristics and outcomes, the former will consequently constitute cues. People may then pick up on whether the cue is guiding them towards their goal or not and adapt their behaviour accordingly. On this view, the robustness of such phenomena is, at least partly, determined by the cue structure in each given setting. In an experiment, we demonstrate that the attraction effect and the default nudge obtain proportionally to how well the manipulated stimulus characteristics predict the superior option. A similar result is found under a more traditional rule learning manipulation. We suggest that the existence of cue-outcome relationships is an (underappreciated) limiting condition for the robustness of behavioural phenomena. We discuss the implications of this perspective for nudging.

## Introduction

We may consider a behavioural phenomenon to be robust if conceptually similar experimental manipulations consistently produce effects on behaviour in the same direction and of comparable size. High robustness is central to discussions of external validity^1^, theory testing^2^ and the replication crisis^3–5^. Discussions on the limits of robustness have, we believe, tended to take a statistical perspective (e.g.^6–8^). Here, we suggest a theoretically motivated limiting condition for robustness and demonstrate it with empirical examples. While it cannot be applied to all behavioural phenomena, we propose that it has relevance for the rather broad class of studies where the experimental manipulation consists of adapting stimulus characteristics or the context in which they are presented.

**Fig. 1:**
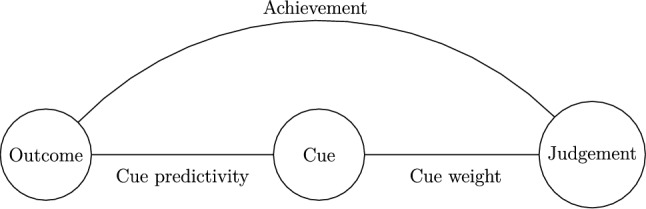
A simplified lens model adapted to the current experiment. There exists some objective association between the distal Outcome and proximal Cue (Cue predictivity). The judge may extract this relationship and adapt their judgement to it (Cue weight). Achievement is how well the judgement corresponds to the outcome.

A natural but, it seems to us, overlooked perspective on robustness can be found in Brunswikian^9,10^ psychology. The “lens model” (Fig. 1) distinguishes between distal, unobserved properties and proximal, observed cues. In any given setting (“ecology”), there exists statistical relationships between the cues and properties. Research in the Brunswikian tradition supposes that the mind is geared towards calibrating its judgement strategies such that it makes the most effective use of the cues to predict the properties (“probabilistic functionalism”,^11^). Cues are, however, typically only imperfect predictors of properties and the challenge for the organism is to learn how much weight to put on each cue. The best one can do is to adapt one’s weighting of cues such that it is equal to how strongly a cue actually predicts a property (i.e. calibrate “cue weights” to be equal to “cue predictivities”,^12^). People appear to be decent at this but performance depends on characteristics of the task and feedback (see^13^ for a review). When cues have a simple (e.g. linear) relationship to the outcome variable (e.g.^14^), this learning is believed to be declarative^15,16^: the extracted contingency can be verbalised, allowing participants to extrapolate beyond the observed cue values.

We tentatively suggest that a theoretical commitment to probabilistic functionalism should make us qualify our expectations of robustness. When experiments involve manipulating characteristics of the options, these manipulations create regularities in the environment that constitute cues. For example, if we manipulate the set of options presented (e.g.^17^), or their attribute values (e.g.^18^), then which kinds of options that co-occur can constitute a cue. If we manipulate the user interface layout (e.g.^19^), then how options are ordered, where they are located on the screen, or which option is pre-selected can constitute a cue. If we manipulate visual characteristics of individual options (e.g.^20^), their shape, colour, or pattern can constitute cues. Participants may then pick up the cue-outcome relationship and adapt their behaviour accordingly. We should thus expect robustness in settings where no such relationships exist, are inferior to some other policy of choice, or where the conditions are too severe to allow adaptation of cue weights. Further downstream, if we believe that phenomena will be robust only in lieu of predictive cue-outcome relationships, then this constitutes a limiting condition on (i) the external validity and replicability^4^ of phenomena and (ii) the scope of the theories they motivate. We tentatively suggest that researchers sometimes over-generalise their findings when they neglect this limitation (e.g.^21,22^).

Apart from this Brunswikian perspective, with its roots in perception psychology, similar thinking has been put forward in social psychology. (We are grateful to an anonymous reviewer for reminding us of this). For example, rather than “cues” and “cue weights”, Schwarz and Bless^23^ speak of “sources of information” providing “routes to judgement”, and “filters” that determine which sources are utilised. Wegener and Petty^24^ emphasised the metacognitive nature of filtering, seeing it as driven by the individual’s beliefs about how different sources of information exerted (undesired) influence on their initial judgement (cf.^25^). This filtering will only be well-tuned in so far as people’s beliefs are accurate and they are motivated and able to filter (see e.g.^26^). This is perhaps the clearest contrast to probabilistic functionalists who instead tend to assume that cue weights will (eventually) be well-calibrated (e.g.^27^) — at least to the extent allowed by the constraints imposed by the environment (cf.^28^). So, while we do not deny that there are nuanced theoretical differences between these traditions, they share the fundamental idea that cue use is adaptive. Despite this recurrent evolution, “this point seems to be strangely both intuitive yet subtle, such that it needs to keep being rediscovered, especially in different subfields” (as phrased by^29^, albeit regarding a different issue).

We do not believe that the perspective described above can be “proven” in any direct sense, much like we do not believe that one can prove that experimental results are, or are not, externally valid *in general* (cf.^30,31^). What one can do, however, is to build a library of cases where it indeed turned out that cue predictivity was a limiting condition for robustness (cf.^32,33^ in the context of external validity). Such demonstrations are of direct relevance to the scrutinised cases, but might also motivate a more general notion of the perspective being important. We submit a contribution to that library here by investigating one famous context effect, one famous nudge and one set up more paradigmatic of the cue learning literature. We will demonstrate that in each case, the paradigmatically expected effect of the manipulation occurs in proportion to the probability with which the manipulated characteristic predicts the outcome of interest. Let us immediately state that this does not imply that previous accounts of these effects are “false”. Rather, it suggests that people are *also* able to adapt to achieve their goals and that this adaptation may determine what strategy is disclosed by a given task (cf.^34^).

The first effect we will be focusing on is the attraction effect. The attraction effect exists when adding some dominated option (a decoy) to an option set increases the probability of a participant selecting the dominating option (the option that has a decoy) rather than a third option that dominates neither the decoy nor the option that has a decoy. This constitutes a violation of independence from irrelevant alternatives, which states that the choice between any pair of options should be unaffected by the addition or removal of other options to/from the option set^35^. Although the attraction effect has been demonstrated many times (e.g.^36–38^), failures to replicate the effect (e.g.^39^) have led to studies qualifying the conditions under which it occurs (e.g.^40–42^). Indeed, sometimes the effect of an option having a decoy is even reversed (“repulsion effect”,^43^). It seems important to tune the differences between options (e.g.^44,45^) such that they exist within some critical zone where the effect can be observed. There are (at least) two prominent psychological explanations of the effect: the decoy could provide individuals with a convenient argument for justifying their choice^46–49^ or it could arise from people evaluating the options through sequential comparisons of their attributes^50,51^.

The second effect we will be focusing on is the default nudge (e.g.^52^). A “nudge”^21^ is a manipulation of the context of a choice that does not restrict the freedom of choice of the individual. A famous example is to make some option the pre-selected default, which should make it more likely to be chosen over the alternatives (e.g.^53,54^). However, in some settings this nudge has yielded null or even counter-predictive effects^55–58^. A controversy surrounding a recent meta analysis of nudges

^59^ has highlighted that the literature also suffers from severe publication bias^60–62^. If some nudges are not robust it could thus be because they simply do not work in the first place.

In a given experiment investigating the attraction effect, it could turn out that the option that has a decoy tends to yield a superior, inferior, or on-par outcome compared to the alternatives. Similarly, in a default nudge study, it could turn out that the default option tends to be a “sensible” default^63^ that yields the superior outcome. Participants may pick up and rely on such tendencies in order to make a choice, selecting for example the default option only in so far as it indeed tends to be the “sensible” choice. We propose that such a reliance can compete with — or even out-compete — the attraction effect and the effect of a default nudge as typically understood. If so, it suggests that the attraction effect and default nudge effect *per se* may only shine through under particular circumstances. Specifically, when the option that has a decoy/the default option tends to yield about as good outcomes as the alternatives — a situation where the participant has reason to be indifferent. We think of these as empirical examples that motivate increased attention to how cue-outcome relationships may limit robustness.

## Method

Participants performed 40 trials of a task previously used to study the attraction effect^64,65^. Each participant was enrolled in one of three cue type conditions: the decoy, default, or rule condition. While we present these as three conditions within a single experiment, one could equally well think of them as three separate experiments. The decoy and default cue types are described in the Introduction. The rule cue type manipulated a physical stimulus characteristic, similarly to traditional cue learning literature (e.g.^66,67^). This was intended as a comparison condition to see if the results from the two former cue types were comparable to this more traditional cue type, potentially illustrating that the former are cues “like any other”. The primary manipulation was how predictive the decoy/default/rule is (the probability of the cue indicating the superior option), which we randomised for each participant individually. We view each cue type condition as a replication of the effect of cue predictivity on choice behaviour.

### Participants

Data was collected on Prolific, a popular online social science laboratory (see^68,69^ for overviews). Data for the decoy and default conditions was collected from September through October of 2021. Data for the rule condition was collected through March of 2024. We had a rolling recruitment with the preregistered goal of having at least 300 valid participants for each cue type. In total, we recruited 1010 participants. After applying our preregistered exclusion criterion of removing any participant who did not complete the experiment within one hour, the final sample contained 302 participants in the decoy condition, 306 in the default condition, and 301 in the rule condition. The average age of these participants was 29.3 years (sd = 10.22) and 47% were women.

Participation was open to all Prolific users who spoke fluent English, had participated in between 10 and 10 000 studies on Prolific, and had an approval rate of at least 95%. We did not specifically recruit from any vulnerable groups. We did not collect any demographic data on indicators of vulnerability due to ethical concerns — such data constitutes sensitive personal data, as per European and Swedish law, and must not be collected unless specifically motivated by the research question.

Participants collected a payoff (the area of their selected option minus its price) in experimental units in each round. After the experiment, the points were converted into GBP according to an exchange rate known to the subjects (1500 units to GBP 1). The average payment was GBP 2.85. The payment was calibrated to meet a fair standard of expected hourly pay.

### Design

**Fig. 2:**
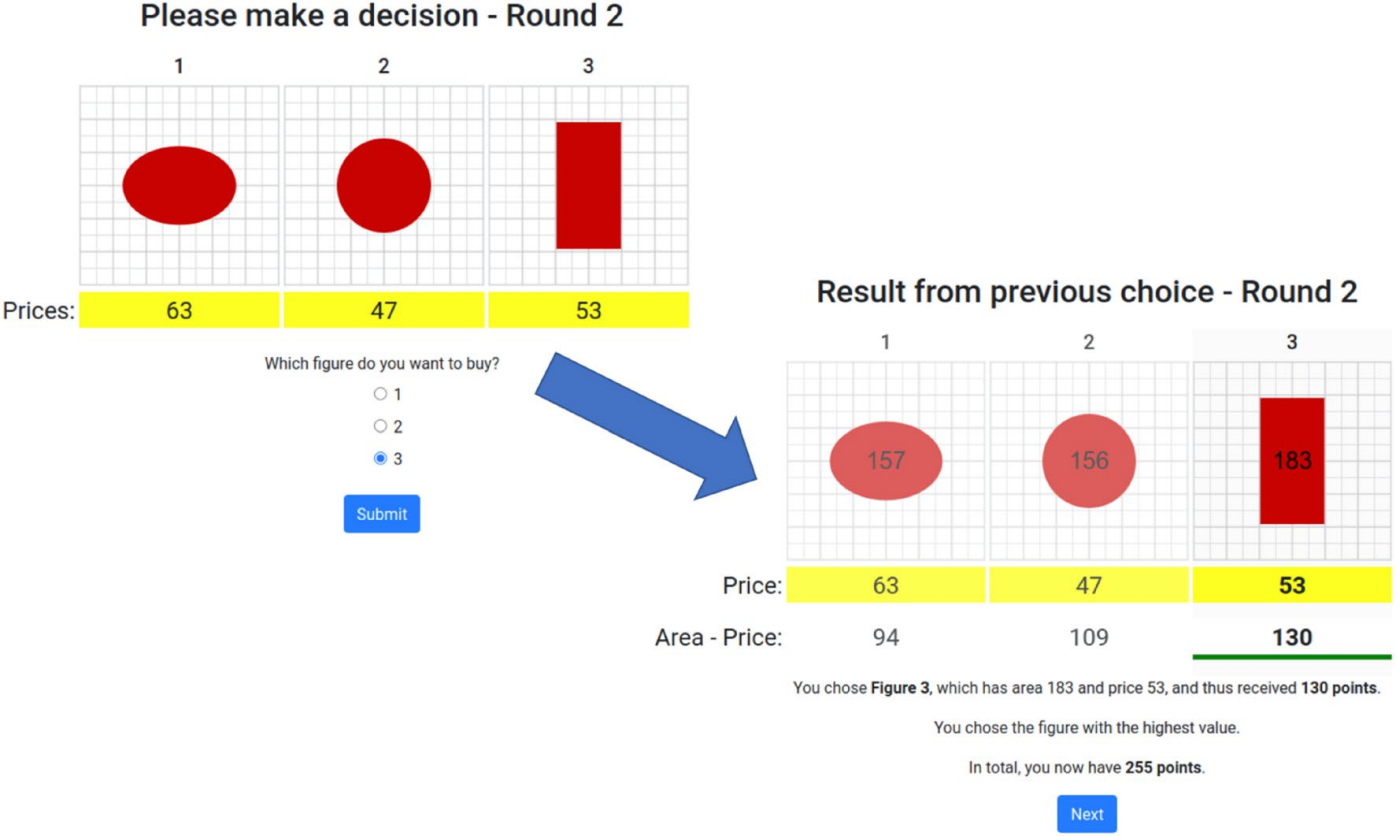
The task as it appeared to participants. A participant in the default condition is about to choose option 3, a rectangle of area 183 and price 53. Once they have done so it is revealed that this yields 130 points. They are also informed that they chose the superior option, how many points the alternatives would have yielded, and their total score.

The task was based on Crosetto and Gaudeul^64^ and Trueblood et al.^65^. Participants were asked to choose one of three geometric shapes (each of which could be either a vertical rectangle, horizontal rectangle, vertical ellipse, horizontal ellipse, square, or circle) with different areas (e.g. 2 cm, 2.5 cm) presented on a grid. Each option came at some price, in experimental units. Upon choosing an option, the participant received a payoff equal to the area minus the price of the chosen option. That is, the immediately available information was sufficient to find the superior option (up to perceptual error); participants could disregard cue predictivity completely and still solve the task successfully. The participant also received full feedback^70^: the areas of all shapes and their corresponding payoffs were presented on screen after each choice. At the end of the experiment, the experimental units were converted into pound sterling (GBP) at a predetermined exchange rate of 1500 units to GBP 1. Participants had to wait at least 5 seconds per trial before they could submit a choice, to invite some consideration. See Fig. 2 for task interface and feedback screen and Appendix A, Fig. S1, for task instructions.

We generalised the design of earlier decoy and default studies by generating option features randomly, from predetermined distributions, as follows: firstly, three payoffs were drawn from the same normal distribution and then ranked. Secondly, the price of each option was drawn from another normal distribution. Each option’s area was determined by adding the payoff and the price. Thirdly, each option’s geometric shape was randomised (but see the Rule condition below). These features exhaustively defined each option. In addition to this shared feature generation protocol, options were adjusted depending on cue type condition as described under the respective subheadings below. We manipulated cue predictivity between participants by randomising the probability of the cue indicating the superior option (“assigned predictivity”). In 28 of the rounds, the cue was generated according to the assigned predictivity (“cue treatment rounds”). In the remaining 12 rounds, the cue was unpredictive in expectation (uncorrelated with payoff) for all participants and identically generated for all participants within the same cue type condition. We used only these 12 test rounds to test our hypotheses, in order to respect measurement invariance^71,72^. Treatment and test rounds were mixed and presented in random order. Option position (left, middle, or right) was randomised. In trials where the cue did not indicate the superior option, it indicated one of the other two options. That is: for each participant, there existed some individual probability that the option that has a decoy/default option/option of a particular shape (see conditions below) would also, on any given trial, be the option with the greatest potential payoff. So, on such trials the option that has a decoy, for example, would also turn out to be the option with the highest payoff (largest difference between area and price). On the rest of the trials, the option that has a decoy would not be the one with the highest payoff. There were no other differences between these two kinds of trials. If participants picked up on that having a decoy/being the default/being of a particular shape was a cue for being the superior option, they could use it to adapt their decision strategy. The probability, the relevant cue type, and, indeed, the existence of predictive cues were never stated to participants — they had to be discovered from experience. There were no visual indications of whether a round was a treatment or test round.

For any given trial, we indicated whether the cue did predict the superior option in that round using a dummy variable:

1 $$\begin{aligned} \text {cue predictive} = {\left\{ \begin{array}{ll} 1 & \text {if superior option has a decoy/is default/is indicated by rule} \\ 0 & \text {otherwise} \end{array}\right. } \end{aligned}$$

We proceeded to calculate the share of such predictive rounds observed by the participant so far. We call that variable historical predictivity:

2 $$\begin{aligned} \text {historical predictivity}_r = \sum _{i=1}^{r-1} \frac{\text {cue predictive}_{i}}{r-1} \end{aligned}$$

where *r* is rounds from 1 to 43, rounds 1 to 3 are practice rounds (see Procedure), and rounds 4 to 43 are the experiment rounds. This measure was the independent variable in our analyses. We thus did not investigate the effect of the *assigned* predictivity (cf. intention to treat,^73^) but of the empirical, historical predictivity that participants actually observed. We now describe how we adjusted the generated options for each cue type condition.

#### Decoy condition

In each of the 28 treatment rounds, option features were randomised (see Design) to generate three options. The generated options were adjusted as follows. With probability *p* equal to the assigned predictivity, the superior option was selected to have a decoy. If so, one of the remaining options (either the second or the third best) was chosen with 50% probability to be the superior option’s decoy. Both the superior option and decoy option - the option that *has* a decoy and the option that *is* a decoy - were made to assume the same randomised shape. This facilitated ranking them by area (see^74–76^), making it slightly easier to identify that the decoy was dominated. The decoy option was then adjusted to be dominated by the superior option in both area and price as follows. Remember, the superior option would at this stage already, by definition, have a higher payoff than the decoy. We could thus calculate a payoff difference between the two. We adjusted the decoy by randomising a proportion [0,1]. That randomised proportion of the payoff difference was added to the superior option’s price to generate the decoy’s price. The rest of the payoff difference was deducted from the superior option’s area to generate the decoy’s area.

With probability $1-p$, the second best option was chosen to have a decoy. If so, the worst option was the decoy and was adjusted as before. In each of the 12 test rounds, the generated options were adjusted as follows. Firstly, either the superior or the second-best option was determined to have a decoy with equal probability. That option was then assigned the worst option as its decoy. The decoy was adjusted as before. In the test rounds, an option having a decoy was thus not indicative of whether it was the superior or second best option but it did indicate that it was not the worst option.

#### Default condition

In each of the 28 treatment rounds, option features were randomised (see Design) to generate three options. The generated options were adjusted as follows. With probability *p* equal to the assigned predictivity, the superior option of the three was pre-selected and participants thus only had to click the “submit” button to choose it. With probability $1-p$, one of the two inferior options was pre-selected. In the 12 test rounds, one of the generated options was pre-selected by default with a uniform probability over the three options. In the test rounds, the default was thus not indicative of whether an option was superior, second best or third best.

#### Rule condition

For the 28 treatment rounds, one geometric shape per participant was randomised to be the “indicator shape” (e.g. square). Option features were randomised as described in Design to generate three options. With probability *p* the superior option was forced to assume the indicator shape and the shapes of the other two options were re-randomised to be any other shape. In $1-p$ of cases, one of the two inferior options was instead forced to assume the indicator shape. The rule was thus that one of the shapes indicated the superior option with some probability *p*, and that shape would be present on every trial whether it was indicative or not. In the 12 test rounds, option features were generated as per Design and a randomly selected option was then forced to assume the indicator shape while the remaining two had their shapes re-randomised. In the test rounds, the indicator shape was thus not indicative of whether an option was superior, second best or third best.

### Analyses, open science notes and ethics

We preregistered the experiment task, sample size, analyses and the hypotheses of positive associations between historical predictivity and following the decoy/default/rule in the test rounds.

To test these hypotheses, and as preregistered, we subsetted the data in the decoy, default, and rule condition, respectively, to obtain only the 12 test rounds per condition. Separately for each condition, we then regressed the binary variable of whether the option with a decoy/default option/option indicated by the rule was chosen on the historical predictivity. For each regression, we predicted a positive coefficient of the historical predictivity measure.

According to the Swedish act concerning the Ethical Review of Research involving humans (2003:460), blanket ethics approval applied to this study. The study was conducted in accordance with the Declaration of Helsinki.

### Procedure

Participants gave informed consent (Fig. S2), received identical instructions in all conditions, and were able to practice the task for three rounds. The practice rounds were generated according to the participants’ respective treatment levels: their cue type and their assigned predictivity. Participants then needed to pass a comprehension quiz with 4 questions (Fig. S3). If they failed it more than 10 times, their participation was terminated. If they passed, participants proceeded through the 40 experiment rounds. After the experiment, participants were compensated for their time.

### Results

We find evidence of positive effects of historical predictivity in our preregistered regressions for all cue types, see Table 1. An increase of historical predictivity by 10 percentage points results in an estimated increase in the probability of choosing the option that has a decoy, the default, and the option indicated by the rule by 1.29%, 1.58%, and 2.21%, respectively.

**Table 1:** Regression parameter values for each dependent variable.

	Dependent variable		
	Option with decoy was chosen	Default option was chosen	Option indicated by rule was chosen
	(1)	(2)	(3)
Constant	0.476	0.283	0.265
	(0.021)	(0.020)	(0.020)
Historical predictivity	0.129	0.158	0.221
	(0.037)	(0.039)	(0.044)

Standard errors in parentheses, robust and clustered on individual level. $p < 0.001$ for all estimates. In Appendix C, Table S1, we show that these results are robust to including participant level random effects instead of the clustered standard errors.

**Fig. 3:**
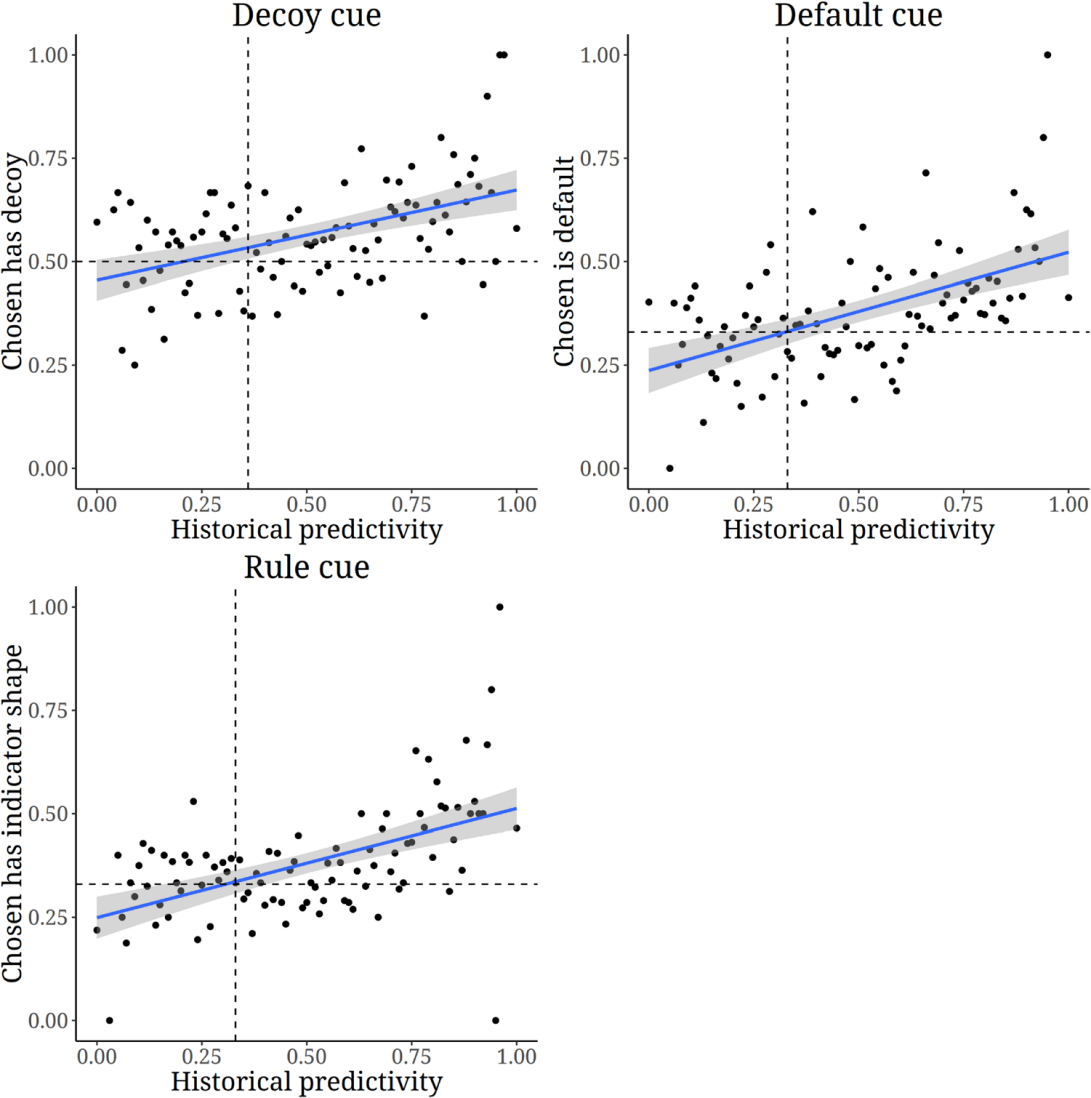
Regressions of historical predictivity on choosing the option that has a decoy/is the default/is of the indicator shape. The latter are binary variables, making individual observations difficult to visualise in a scatterplot since they will all sit at 0 or 1. We therefore group all observations by historical predictivity rounded to two decimal places. Each marker is thus a local average for a small subset of observations at each level of historical predictivity. Intersections of dashed lines indicate at what level of cue predictivity choice probabilities should be at chance if participants respond to the cues (see Appendix B, Fig. S4,). The regression lines should thus intersect these intersections.

We can graph the estimated linear relationship between the mean probability of choosing the option that has a decoy/is the default/is indicated by the rule and the historical predictivity, see Fig. 3. What the relationships should look like in the presence or absence of an adaptation to cue predictivity differs slightly between conditions. We walk through those predictions in Appendix B and state the take-home message here: these graphs show that when the cue has historically been predictive, participants choose the option that has a decoy/is the default/is indicated by the rule above chance frequency. When the cue has not been predictive, there is a tendency towards a slight attraction effect but choice in the default condition is on average practically random. When the cue has been predictive of which option *is not* superior, the superior option is on average chosen below chance frequency. Indeed, if we follow the convention of evaluating these effects based on group averages (contrast this to^77^) we can find cross sections where the effects appear, disappear, and (less clearly) are reversed.

The estimated cue weight (coefficient of historical predictivity, Table 1) appears to be greatest for the rule condition, second greatest for the default condition, and smallest for the decoy condition. A tentative speculation is that this reflects how complex the cues are: the rule involves a property of the stimulus, the default involves an association between the response mode and the stimulus, while the decoy involves a relationship between two stimuli. The reliance on cues could be moderated by how easy they are to pick up.

## Discussion

Our most fundamental conclusion is that the prevalence of the effects investigated here scaled with how strongly the cues provided by characteristics of the experimental manipulations had predicted the superior option. Less clearly, when the manipulations helped participants predict which option was *not* superior, the directions of the effects seemed to reverse. The effects we focused on here were thus not robust to cue-outcome relationships. As an anonymous reviewer pointed out, a probabilistic functionalist^11^ or (social psychological) judgement correction researcher (e.g.^23,26,24^) might find these results highly expected; established principles applied to a novel setting. We agree! Despite this, these established principles seem (to us) to not have permeated into the broader social scientific and meta-scientific discourse. It does not seem recognised that they can limit robustness in the way they did here. We tentatively suggest that this perspective may be important for such discussions.

One way to interpret the results is that cue predictivity is a potential confound of the substantive effects. If so, the question becomes how to avoid the confounding. We would perhaps prefer a different interpretation: all effects are contextual in the sense that they are moderated by other variables than the main variable of interest (cf.^6^). There is no pure or “true” effect of *X* on *Y*, only a conditional effect $Y = f(X_1|X_2,X_3,...,X_n)$ (cf.^78^). A robust effect would be one where these moderators $X_2, X_3,...,X_n$ exert only a minor influence on the final result. Here we have demonstrated a moderator that has appreciable influence, but the solution is not to try do to away with it but to embrace it. Any investigation of, say, the attraction effect will reveal that effect *conditional on a particular level of cue predictivity*. That is absolutely fine, as long as we interpret the results in that manner and carry that interpretation with us as we draw theoretical and applied conclusions. If we have some context in mind that we want our results to hold in, we should set the cue predictivities in our study such that they are representative of that setting (cf. “representative design”,^79,80^).

Given that view, what do these results mean for the literatures on the attraction effect and default nudge? Firstly, the sometimes failed attempts at replication of the attraction effect (see Introduction) indicate that whatever cognitive mechanism explains it is not ubiquitous. While we encourage independent replications, if we take the present results as read they further circumscribe such a mechanism: a theory that explains the attraction effect (see e.g. General discussion in^65^) will not be a theory of “how people make choices” but rather of “how some people make choices, at least when there are no highly predictive cues”. To be very clear: this does not make such theories less interesting, exciting, or worthwhile. However, we agree with Regenwetter, Robinson, and Wang^81^ that theoretical scope should be made explicit. Secondly, effects of defaults have been proclaimed to be due to people using a “default heuristic” (e.g.^82^). Labelling something a “heuristic” is, on its own, a redescription rather than an explanation (cf.^83^) but in this case the suggestion seems to be that there exists some more or less hard wired algorithm that people are wont to use (see^84^). We suggest that it might be more fruitful to proclaim the default a “cue” and (re)describe default effects as the cue having a “high cue weight” because this situates the phenomenon within a principled theoretical framework. Such a framework might then open up for new explanations. Specifically that, far from being hard wired, default effects are emergent properties of the individual attuning to their environment. Indeed, here we saw no default effect at all on average when historical cue predictivity was about chance level (Fig. 3). It might also open up new avenues of empirical inquiry. For example, Brunswikian psychology has a long history of lens model analysis (e.g.^85,86^) where cue predictivity, cue weight, and achievement are estimated empirically for a given setting. Such an exercise sheds light on both the normative (cue predictivity) and empirical (cue weight) solution, as well as raw performance (achievement).

One could interpret these results in light of the literature on probability learning. A now classic review by Peterson and Beach^87^ presented people as “intuitive statisticians” who are able to learn statistics of observed samples of outcomes (see e.g.^88,2,89–91^ for contemporary work). When making discrete decisions based on such experience, participants are often found to “probability match”^92–95^ - they select the superior option proportionally to how often it yielded the superior outcome. This deviates from the normatively optimal strategy of “maximisation” - to always select the option most likely to yield the superior outcome - but has been argued to be superior in an ecology where seemingly stochastic payoffs may in fact reflect sequential dependencies^96^. One could interpret Fig. 3 as showing a tendency towards probability matching here too. That being said, such a discussion is not central to our present claim: it is the *function* rather than the *mechanism* (see Tinbergen’s levels of analysis,^97^) that motivates our limiting condition for robustness.

What are the limiting conditions of this limiting condition? First and foremost, cue-outcome relationships must exist and be strong enough to be picked up. Mere repetition is not sufficient. People can learn cue weights or make one-shot decisions using inferred ones, and it does not seem to matter much if the relevant cues are stated explicitly by an experimenter or if the individual has to extract them without guidance^13^, as in this study. Secondly, relying on the cue must improve on other grounds for choice (cf. “cue family hierarchy”,^98,99^). In the present experiment, participants were provided with all the information necessary to, at least in principle, always identify the superior option. Had we made the task very easy, by greatly increasing the differences in area and price between options, a probabilistic functionalist would expect participants to ignore the cue and instead base their decision purely on the option attributes. Thirdly, we agree with flexible correction model theorists (e.g.^26,24^) that one must be motivated to achieve the outcome a given cue predicts for it to affect choice behaviour. However, we emphasise that those goals can be highly idiosyncratic (e.g.^100^), although incentivisation can reduce that heterogeneity (e.g.^101^).

Whether the limiting condition we have argued for here is an important one hinges on there existing cue-outcome relationships in the settings where we “want” phenomena to be robust (cf.^1^). We tentatively believe such settings to exist. Nudges provide a case in point (see also^102^). The nudge will, again, involve the creation of cues. It will be applied with the aim of moving individuals towards making a particular choice which begets a particular outcome. This constitutes a cue-outcome relationship. When the choice architect tries to nudge the individual towards an outcome that is not aligned with the latter’s goals, the individual may eventually recalibrate their cue weights to disregard the nudge. Our limiting condition could thereby come into play here; it could be that nudges have more robust effects when they nudge an individual *towards* her self-perceived goals (“motivational alignment”,^103^). On the flip side, this limitation could also be a boon. Authorities and so-called “nudge units” could potentially exploit people’s capacity to adapt to cue-outcome relationships to “boost”^104^ individuals in at least two ways. Firstly, they could ensure motivational alignment between nudgers and nudgees and then construct highly predictive cues for those aligned goals. Alternatively, they could ensure that there exist highly predictive cues for a diverse range of outcomes. That way, one might not need to infer the individual’s self-perceived goals. Instead, one might trust individuals to pick up on the relevant cues and rely on them to when making their choices. In Brunswikian terms, this would allow individuals to obtain high achievement (Fig. 1) to whatever outcome they desire. While speculative, we believe this to be an exciting prospect for future research to address.

## Supplementary Information

Supplementary Material 1

## Data Availability

The decoy and default conditions were preregistered at https://doi.org/10.17605/OSF.IO/DS4XP. Data for the rule condition was collected later and preregistered at https://doi.org/10.17605/OSF.IO/852ZV. A preregistered pilot experiment is available in Mandl (2022). All data, materials and analysis code are available at https://doi.org/10.17605/OSF.IO/J362H.
