# Supplementary material for: Probabilistic functionalism as a limiting condition for robustness
Source: Sci Rep. 2025 Dec 22;15:44333. doi: 10.1038/s41598-025-32580-z (PMC12728183; doi:10.1038/s41598-025-32580-z)
Supplement: Supplementary file 1 — Supplementary Material 1 [file 41598_2025_32580_MOESM1_ESM.pdf]

Appendix for “Probabilistic functionalism as a limiting condition for robustness”

Mattias Forsgren[1], Benjamin Mandl[2,3], Gustav Karreskog Rehbinder[4,5]

1. Department of Psychology, Uppsala University, Sweden
2. Department of Economics, Stockholm School of Economics, Sweden
3. Verbund AG, Austria
4. Institute for Future Studies, Sweden
5. Department of Economics, Uppsala University, Sweden

Appendix A: Consent, experimental instructions and comprehension quiz

Please find the experimental instructions, the consent page, and the comprehension quiz below. The default and decoy condition data were collected when the third author was affiliated with the Swedish School of Economics, while he had moved to Uppsala University by the time that the rule condition data were collected. The logo and affiliation displayed at the top of the informed consent form thus differed between these conditions. Blank spaces between lines in the consent form have been reduced to fit it on a single page.

Instructions

Welcome to this experiment on decision-making. Please read the following instructions carefully. The experiment takes about 15 minutes and consists of 40 rounds of the task. You will earn points by performing well in the task. Your total payment will be determined at the end of the experiment by the number of points you earned. The conversion rate between GBP and points is £1 per 1500 points. This payment includes the guaranteed £2.

Instructions

Background

Imagine you are buying paint to paint a large surface. You go to the store to buy supplies. At the store, you can choose between three buckets of paint. The area that can be covered with a given bucket is presented as shapes. Your goal is to get the best deal for the paint: you want to be able to paint as large a surface as possible per dollar. After you have used your bucket of paint, you return to the store to buy more paint, for 40 rounds.

- In each round you will see three shapes with prices below them
- Your task is to choose the option with the largest difference between area and price
- Your points per round are equal to the area of the chosen figure minus the price of the figure

Example

In each round, you are asked to choose one of three figures. See an example below:

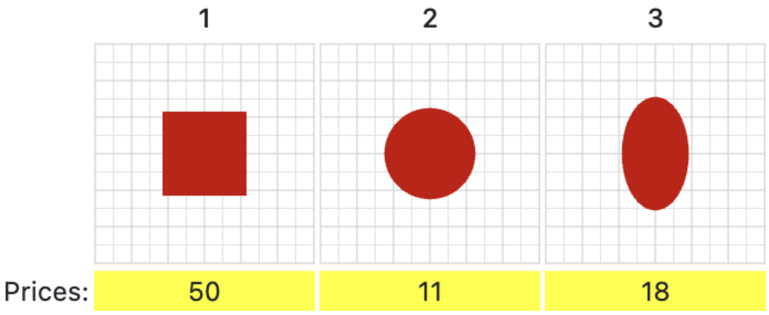

- If option 1 has an area of 150 and a price of 50, you receive 100 points if you choose this option
- The area of the canvas is 900, so all shapes you will see have an area below 900

Additional rules

You are required to spend at least 5 seconds on each decision before you can submit an answer.

Practice and Quiz

You will now practice the task for three rounds. The points from your practice rounds will not be saved and do not affect your final payoff. After the practice, there will be a short quiz about the task that you will need to pass to proceed to the experiment. Please click on the submit button to start.

Submit

Figure S1

Instructions

INFORMED CONSENT FORM

Principal investigator: Gustav Karreskog Rehbinder

Uppsala University

UPPSALA
UNIVERSITET

Welcome to this study about decision-making.

We are asking you to participate in a research study. This form will give you some information about the study. Please read the form carefully before proceeding.

A. Project Description

1. This study will ask you to choose alternatives from a list of options to maximize your reward.
2. The estimated time to complete this study is 15 minutes.
3. The research is being conducted with the goal of publication in academic journals and presentation at academic conferences.

B. Risks and Benefits

Your participation in this study does not involve any physical or emotional risk to you beyond those of daily life. None of the information presented is designed to be upsetting or offensive in any way. The potential benefit of the study is a better scientific understanding of the psychological underpinnings that govern human judgement.

C. Compensation

At the end of the session, you will be compensated depending on the decisions you make in the experiment. If you fail a simple comprehension quiz you might not be compensated at all. Estimated hourly wage is between 8£ and 15£.

D. Confidentiality

To secure the confidentiality of your responses, your name and other identifying information will never be attached to your answers. All data collected will be analyzed in aggregate form. Spreadsheets on which the data are entered will contain no identifiable information. Your privacy will be maintained in all published and written data resulting from this study.

E. Contacts

If you have any questions or concerns about this study, you may use the Prolifc message function or contact Gustav Karreskog Rehbinder at gustav.karreskog@nek.uu.se

Do you wish to participate in this study?

Yes No

Figure S2

Consent page

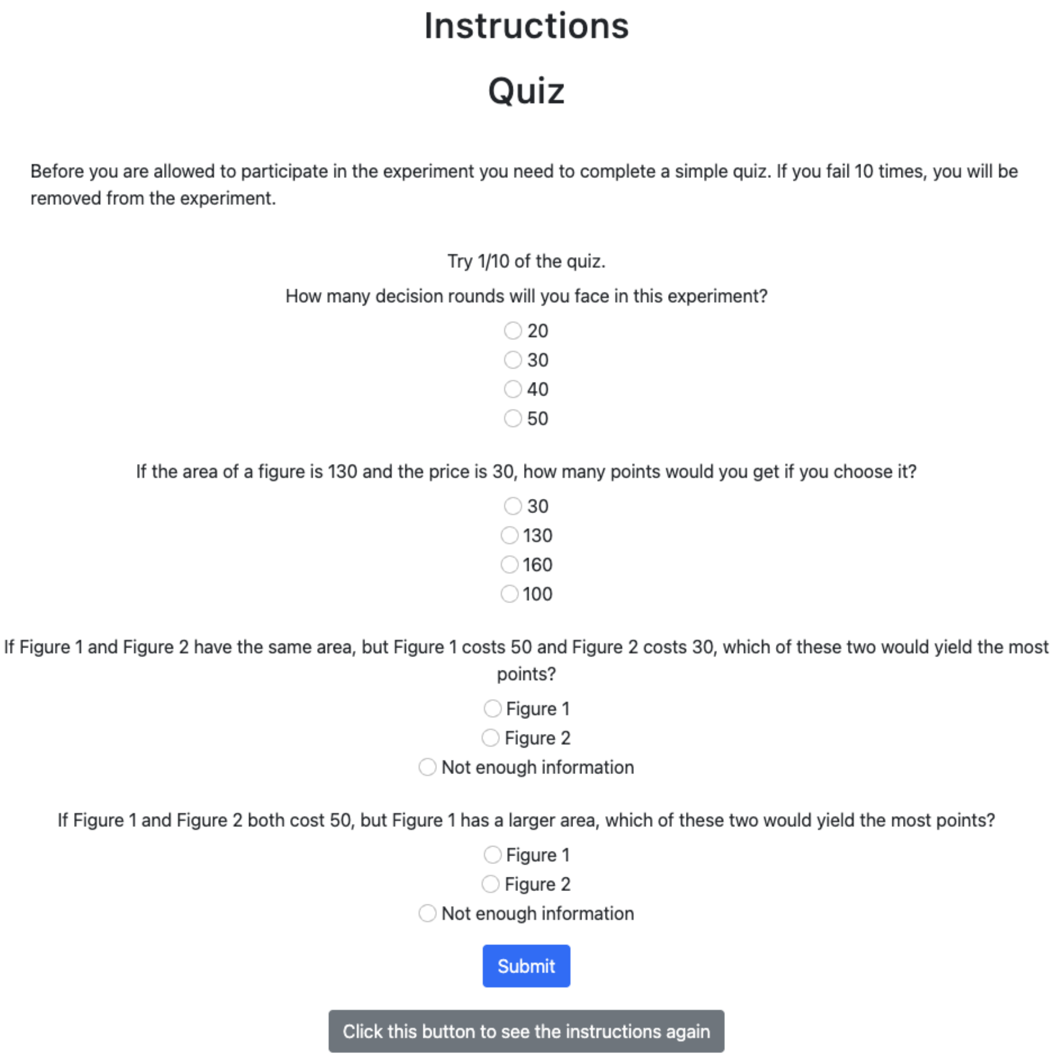
Instructions

Quiz

Before you are allowed to participate in the experiment you need to complete a simple quiz. If you fail 10 times, you will be removed from the experiment.

Try 1/10 of the quiz.

How many decision rounds will you face in this experiment?

- 20
- 30
- 40
- 50

If the area of a figure is 130 and the price is 30, how many points would you get if you choose it?

- 30
- 130
- 160
- 100

If Figure 1 and Figure 2 have the same area, but Figure 1 costs 50 and Figure 2 costs 30, which of these two would yield the most points?

- Figure 1
- Figure 2
- Not enough information

If Figure 1 and Figure 2 both cost 50, but Figure 1 has a larger area, which of these two would yield the most points?

- Figure 1
- Figure 2
- Not enough information

Submit

Click this button to see the instructions again

Figure S3

Comprehension quiz

Appendix B: Intersections of dashed lines in Figure 3 in the main body

Figure 3 in the main body displays dashed lines that the regression lines should intersect if participants respond to the cues. Here, we walk through how the positions of these intersections are obtained.

For the default and rule conditions it is quite straightforward. Remember, option payoffs are randomised. If the participants ignore the cue, the shape that is the default/is of the indicator shape should be chosen 1/3 of the time in the test rounds. If the participants respond to the cue, the shape that is the default/is of the indicator shape should be chosen 1/3 of the time only when historical predictivity is 1/3 and increase with historical predictivity. This is indeed the pattern we see in Figure 3 in the main body.

For the decoy effect, understanding what relationship we should obtain is slightly more intricate. First note that the option that has a decoy is never the worst option and that the option that is a decoy is never the best option. If a participant observes this, they should never choose the option that is a decoy, effectively reducing the task to a choice between two options (the option that has a decoy and the third, remaining option, see also [1]). Considering those two options, the option that has a decoy can only ever be the best or second best option (since the option that is a decoy is always dominated by the option that has a decoy). The remaining option however (which neither has a decoy nor is the decoy), can be the best, second best, or worst option. This means that even if the option that has a decoy is the best option only half of the time, it might still have a higher expected payoff than the remaining option.

We want to find the level of historical predictivity at which we should expect participants to choose the option that has the decoy about 1/2 of the time, when responding to the above put not historical predictivity per se. Thus, we must find the point where the average payoffs of choosing the option that has a decoy and the option that neither has, nor is, a decoy are equal. We do so by regressing historical predictivity on average payoff for each of the two kinds of options separately and finding the level of historical predictivity where the

two are equal. As is evident from Figure S4, this occurs when historical predictivity ≈ 36%. Thus: if participants ignore the decoy cue's historical predictivity but observe that the option that is a decoy is never superior, the option that has a decoy should thus be chosen 1/2 of the time. If participants respond to the decoy cue, the option that has a decoy should be chosen 1/2 of the time only when historical predictivity is 36% and increase with historical predictivity.

Do these expected patterns emerge in the data? For the default and rule conditions, the regression line intersects 1/3 when historical predictivity is about 1/3. For the decoy condition, the regression line intersects 1/2 a bit too early. However, it is still quite close to 1/2 when historical predictivity is about 36% and (Figure 3 in the main body). This is consistent with a separate contribution from an attraction effect as traditionally envisioned [2, 3] being added to an effect of historical predictivity, bumping the regression line upwards without changing its slope. All in all, we seem to observe relationships consistent with participants responding to historical cue predictivity.

Appendix C: Participant level random effect instead of clustered standard errors

As preregistered, we reported regressions with standard errors clustered on the participant level. We realised that an alternative approach would have been to instead include participant level random effects. We do so here (see Table S1) and find that this does not change the qualitative results, although the coefficients for historical predictivity are indeed slightly smaller than those from the preregistered analyses.

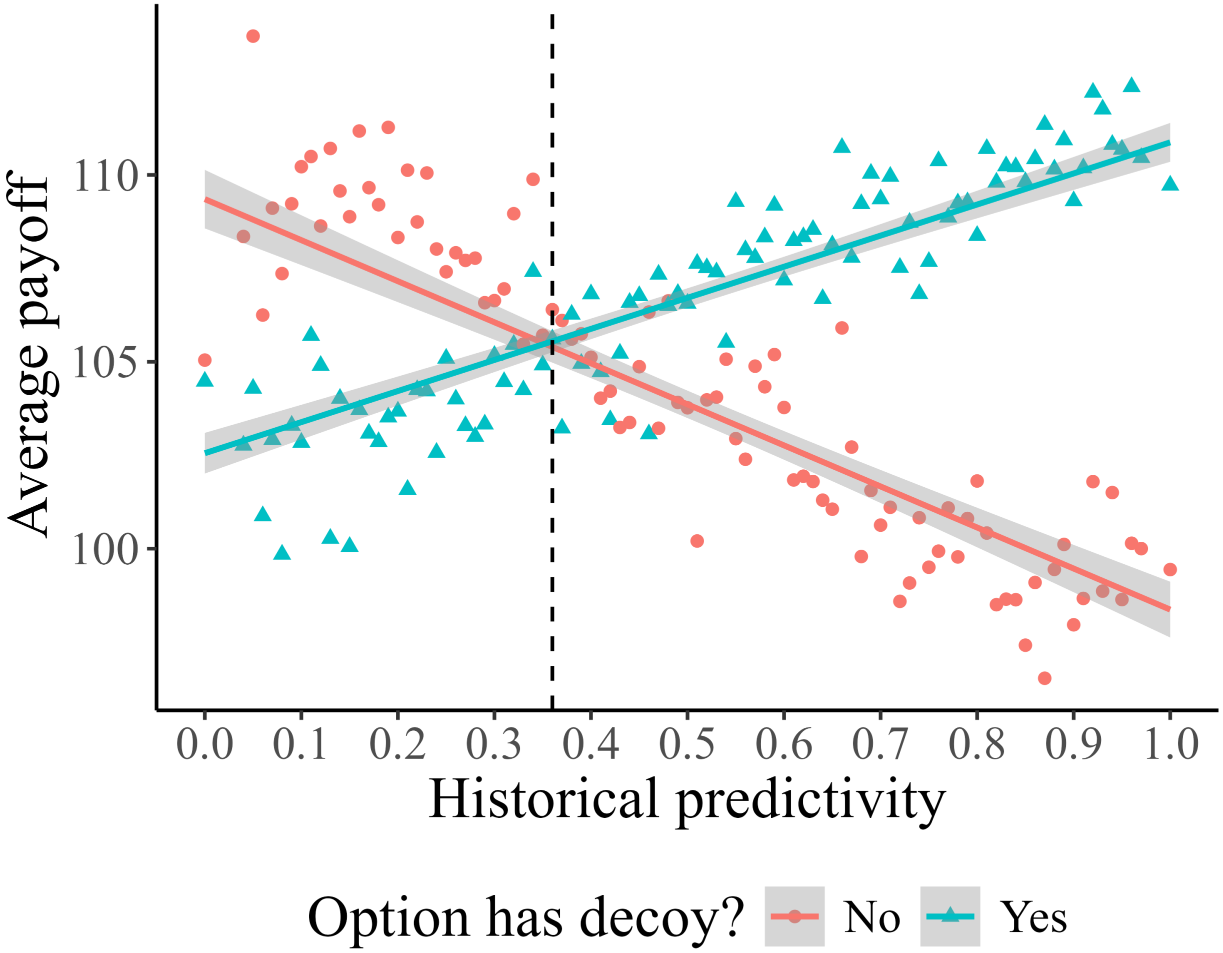

Figure S4

Regression lines of historical predictivity on average payoff for options that have a decoy and options that neither have, nor are, a decoy. Vertical dashed line indicates approximate intersection.

Table S1

Alternative regression parameter values for each dependent variable.

	Dependent variable		
	Option with decoy was chosen	Default option was chosen	Option indicated by rule was chosen
	(1)	(2)	(3)
Constant	0.478	0.289	0.274
	(0.021)	(0.020)	(0.021)
Historical predictivity	0.124	0.145	0.203
	(0.036)	(0.036)	(0.039)

Note: Alternative regressions with participant level random intercepts instead of clustered standard errors. $p < 0.001$ for all estimates.

References

[1] Paulo Natenzon. "Random choice and learning". In: *Journal of Political Economy* 127.1 (2019), pp. 419–457. ISSN: 1537534X. DOI: 10.1086/700762.

[2] Robert M. Roe, Jermone R. Busemeyer, and James T. Townsend. "Multialternative decision field theory: A dynamic connectionst model of decision making." In: *Psychological Review* 108.2 (2001), pp. 370–392. DOI: 10.1037/0033-295x.108.2.370. URL: https://doi.org/10.1037/0033-295x.108.2.370.

[3] Jennifer S. Trueblood, Scott D. Brown, and Andrew Heathcote. "The multiattribute linear ballistic accumulator model of context effects in multialternative choice." In: *Psychological Review* 121.2 (Apr. 2014), pp. 179–205. DOI: 10.1037/a0036137. URL: https://doi.org/10.1037/a0036137.
